# Elimination of detached *Listeria monocytogenes* from the biofilm on stainless steel surfaces during milk and cheese processing using natural plant extracts

**DOI:** 10.1038/s41598-024-52394-9

**Published:** 2024-01-27

**Authors:** Yasmine N. A. El-sawy, Ayah B. Abdel-Salam, Hemmat M. Abd-Elhady, Khadiga A. A. Abou-Taleb, Rania F. Ahmed

**Affiliations:** 1 Agricultural Microbiology Department, Faculty of Agriculture, Ain Shams University, Hadayek Shubra, Cairo, 11241 Egypt; 2 Food Hygiene and Control Department, Faculty of Veterinary Medicine, Cairo University, Giza, 12211 Egypt

**Keywords:** Biotechnology, Cell biology, Microbiology

## Abstract

Bacterial cells can form biofilm on food contact surfaces, becoming a source of food contamination with profound health implications. The current study aimed to determine some Egyptian medicinal plants antibacterial and antibiofilm effects against foodborne bacterial strains in milk plants. Results indicated that four ethanolic plant extracts, Cinnamon (*Cinnamomum verum*), Chamomile (*Matricaria chamomilla*), Marigold (*Calendula officinalis*), and Sage (*Salvia officinalis*), had antibacterial (12.0–26.5 mm of inhibition zone diameter) and antibiofilm (10–99%) activities against *Staphylococcus aureus*, *Bacillus cereus*, *Listeria monocytogenes* and *Salmonella Typhimurium*. The tested extracts had minimum inhibitory concentration values between 0.14 and 2.50 mg/ml and minimum bactericidal concentration values between 0.14 and 12.50 mg/ml. *L. monocytogenes* was more sensitive for all tested ethanolic extracts; Sage and Cinnamon showed a bacteriocidal effect, while Chamomile and Marigold were bacteriostatic. The ethanolic extracts mixture from Chamomile, Sage, and Cinnamon was chosen for its antibiofilm activity against *L. monocytogenes* using L-optimal mixture design. Gas chromatography and mass spectrometry analysis showed that this mixture contained 12 chemical compounds, where 2-Propenal,3-phenyl- had the maximum area % (34.82%). At concentrations up to 500 µg/ml, it had no cytotoxicity in the normal Vero cell line, and the IC_50_ value was 671.76 ± 9.03 µg/ml. Also, this mixture showed the most significant antibacterial effect against detached *L. monocytogenes* cells from formed biofilm in stainless steel milk tanks. At the same time, white soft cheese fortified with this mixture was significantly accepted overall for the panelist (92.2 ± 2.7) than other cheese samples, including the control group.

## Introduction

Biofilm is an architectural colony of microorganisms within a matrix of extracellular polymeric substances (EPS) they produce. Biofilm contains microbial cells adherent to one another and a static surface (living or non-living)**.** Biofilms are found extensively on moist surfaces, such as food, food processing equipment, water pipelines, industrial piping, medical devices, pathological human tissues, and organs^1^. Biofilms formed by *Bacillus licheniformis, Streptococcus uberis, Pseudomonas fluorescens, Pseudomonas fragi, Listeria monocytogenes,* and *Serratia liquefaciens* on the internal surfaces of raw milk tankers may be sources of proteolytic enzymes. The persistence of these microorganisms, especially *Pseudomonas* spp., with the production of thermostable proteases, can lead to the potential spoilage of milk during the subsequent steps in the dairy supply chain^2^**.** Multidrug-resistant microorganisms that arise from the inappropriate use of antibiotics have become a great challenge for public health worldwide, mainly for antibiotic-resistant pathogens^3^. Antimicrobial resistance is now rising to dangerously high levels in all parts of the world, threatening the treatment of an ever-increasing range of infectious diseases. This has become a severe public health problem, primarily due to their ability to form biofilms^4^. Contamination of milk and dairy products with such bacteria could significantly threaten consumer health because of the possibility of antibiotic resistance genes transmission from one pathogen to another. In addition, current antiinfective therapies have low efficacy in treating biofilm-related infections, leading to recurrence, chronicity, and increased morbidity and mortality^5^.

*L. monocytogenes* in dairy plant areas as an environmental contaminant or as a biofilm in plant equipment and utensils is a risky source of biological hazard in the final products^6^. *L. monocytogenes* is not a spore-forming bacteria; it has been reported as the most heat-resistant one^7^. Several strategies have been applied to control *L. monocytogenes* biofilms. Different disinfectants, such as sodium hypochlorite, hydrogen peroxide 2%, and benzalkonium chloride 200 ppm, could not remove listerial biofilms thoroughly. Also, non-thermal technologies such as high-pressure processing, pulsed electric fields, UV light within the range of 10–400 nm, and ultrasound have been explored as alternatives to heat treatment for controlling *L. monocytogenes* in milk and dairy products, but it is inapplicable due to high cost, inadequate process controls and not regulatory approved. Therefore, it is necessary to search for innovative strategies/antibacterial agents capable of overcoming the limitations of conventional antibiotics^4^**.**

Natural compounds, particularly those obtained from plants, have exhibited promising properties in this field. Plant secondary metabolites can act as antibiofilm agents through different mechanisms of action (inhibition of quorum-sensing, motility, adhesion, and reactive oxygen species production, among others) against pathogens, for example, Chamomile, clove, sage plant, and Miswak. These bioactive compounds are present in all plant material like roots, stems, leaves, flowers, fruits, and seeds. Combining different phytochemicals and antibiotics has revealed synergistic or additive effects in biofilm control^4^. Sarghaleh, et al.^8^ stated that plants were used extensively as food (flavoring agent) and medicine. One of the primary attributes of plants is their antibacterial activity, which the industry can utilize to minimize the usage of chemical additions that pose a health risk. The plant *Prangos ferulacea* possesses strong antibacterial properties, particularly against *L. monocytogenes* and gram-positive bacteria. Five medicinal plants (sumac, tamarind, rosemary, roselle, and lemon) were evaluated against six microbial pathogens (*E. coli*, *P. aeruginosa*, *B. subtilis*, *S. aureus*, *Penicillium* sp., and *Aspergillus niger*). These extracts can be applied in substitutes for styptic antibacterial substances. The growth inhibition zone measured 14–45 mm. The growth inhibition zones of the tamarind and roselle extracts were 8–36 and 8–34 mm, respectively^9^. *In* vitro, antibacterial and antibiofilm action has been demonstrated for plant extracts and plant-derived chemical compounds such as essential oils, flavonoids, and terpenoids. Secondary metabolites and other peptidic chemicals from bacteria are also antibiofilm^10,11^. There are broadly five classes of natural compounds (phenolics, essential oils, terpenoids, lectins, alkaloids, polypeptides, and polyacetylenes) with high anti-biofilm properties. Phenolic compounds (phenolic acids, quinones, flavonoids, flavones, flavonols, tannins, and coumarins) act on biofilm by six main mechanisms like substrate deprivation, membrane disruption, and binding to adhesin complex and cell wall; bind to proteins; interact with eukaryotic DNA; and block viral fusion^12,13^. The objective of this study was to explore the antibacterial properties of ethanolic extracts from medicinal plants, with a focus on inhibiting the growth of harmful foodborne pathogenic bacteria that form biofilms. Additionally, the research aimed to evaluate the cytotoxic activity of selected ethanol plant extracts, identify their phytocomponents using gas chromatography and mass spectrometry (GC–MS), and investigate the application of a blend of these plant extracts on stainless steel milk tank surfaces. The goal was to prevent biofilm formation and bacterial shedding during the cheese manufacturing process.

## Materials and methods

### Bacterial pathogens strains and inoculum preparation

The pathogenic bacteria used in this investigation devised from Microbiological Resource Centers (MIRCEN), Cairo, Egypt, and represented *Staphylococcus aureus* ATCC5638, *Bacillus cereus* ATCC11778, *Listeria monocytogenes* ATCC7646, and *Salmonella Typhimurium* ATCC25566.

To prepare standard inoculum, 4–5 colonies of a pure tested pathogenic bacterial culture were picked from an agar plate after 24 h of incubation, subcultured into a tube containing 4 ml of Müller-Hinton broth (OXOID, CM0405 Basingstoke, Hampshire, England)), and then incubated at 37 °C until it reached the turbidity of 0.5 MacFarland standard after 24 h of incubation^14^. The standardized inoculum of the tested bacteria was 2 × 10^7^ colony-forming units (CFU)/ ml.

### Medicinal plant samples collection

Four different plant materials, including Cinnamon (*Cinnamomum verum*) bark, Chamomile (*Matricaria chamomilla*) flowers*,* Marigold (*Calendula officinalis*) flowers, and Sage (*Salvia officinalis*) leaves, were collected from the botanical farm in the Faculty of Agriculture, Ain Shams University, Cairo, Egypt. The plants were washed in water to eliminate particle debris from the surface, and then they were allowed to be air-dried for 7 days at room temperature (28 ± 2 °C). To produce a fine powder, the dried pieces were milled and sieved. The dried plant material was kept at − 20 °C until used.

### Preparation of ethanolic medicinal plant extracts

Twenty-five grams of dried plant powder were extracted separately with 200 ml of 70% ethanol (v/v) (PIOCHEM laboratory chemicals, Giza, Egypt ) in a continuous shaker at 40 °C for 48 h. The resulting ethanol extract was then filtered using filter paper No. 1 Whatman®qualitative filter paper, Grade 1, circles, Sigma-Aldrich) and concentrated in an oven at 40 °C, as the method described by Yeasmen and Islam^15^. To prepare the suspension, the extract was dissolved in 50% (v/v) of dimethyl sulfoxide (DMSO) (Sigma-Aldrich, D2650, Purity; 99%, India) at pH 7.4 and then diluted with sterile distilled water for usage^16^.

### Evaluation of the antibacterial activity of medicinal plant extracts by disc diffusion method

The agar disc diffusion method was used to test the antibacterial effects of ethanolic plant extracts on Mueller Hinton Agar (OXOID, CM0337 Basingstoke, Hampshire, England) plates. One hundred microliters of the prepared standard inoculum of tested cultures were used to culture these plates. Sterilized 6 mm diameter Whatman No.1 filter paper discs (Whatman®qualitative filter paper, Grade 1, circles, Sigma-Aldrich) were placed one at a time onto the surface of the inoculated agar plate, each one saturated with 100 μl of the ethanolic plant extracts. All discs were dried at room temperature overnight. The plates were incubated for 24 h at 37 °C. The diameters of the inhibition zones (mm) were measured around the discs in each plate^17^. Amoxicillin-clavulanic antibiotics (positive control) and DMSO (negative control) were used to compare their antibacterial efficacy with the plant extracts.

### Determination of minimum inhibitory concentration (MIC) of ethanolic plant extracts by Resazurin-based microtiter dilution assay (RMDA)

The MIC was determined using the RMDA on sterile 96 well plates described by Elshikh, et al.^18^. First, the Resazurin dye solution (Canvax™, Spain) was prepared following the procedures provided by Teh, et al.^6^. Then the experiment was performed under aseptic conditions. All wells of microtitre plates (Biologix, China) were filled with sterilized 100 μl of tryptic soy broth (TSB) (HiMedia Laboratories Pvt.Ltd., M011, India). Except for the wells in column code C1 (negative control 1), a ten microliter of diluted standardized inoculum (turbidity adjusted to 0.5 McFarland standard) was added to each microtiter plate well. In contrast, column code C2 contained only standardized inoculum (negative control 2). In columns with code C3, 100 μl of amoxicillin-clavulanic Antibiotic (1.5 mg/ml) was added, then serially diluted two-fold until the last well, and 100 μl was discarded from the previous well (positive control). Two-fold serial dilution of the ethanolic plant extracts (100 μl of Chamomile, Marigold, Sage, and Cinnamon) was added in column codes E1 to E4, respectively. Ten microliters of resazurin dye solution were added to each microtiter plate well and incubated at 37 °C for 24 h. After incubation, the colour changed from violet to pink; pink indicates viable cells, while purple indicates no viable bacteria (dead cells). The MIC value was defined as the lowest concentration of ethanolic plant extracts at which no colour change occurred.

### Determination of minimum bactericidal concentration (MBC)

The MBC was determined by streaking from each well of the MIC plate onto tryptic soy agar plates and incubating at 37 °C for 24 h. MBC was identified as the lowest concentration of extract that did not show any formed colony^19^.

### The action of ethanolic plant extracts

The ratio of MBC/MIC can describe the action of ethanolic plant extracts. When the ratio equals 1 or 2, it means bactericidal effect; when the ratio is ≥ 4 or 16, it means bacteriostatic effects^18^.

### Evaluation of biofilm formation and inhibition

The biofilm formation inhibition capabilities were evaluated according to the modified protocol described by Bhandari, et al.^19^. Briefly, each bacterial strain was grown in TSB containing 1% glucose overnight. The culture was diluted until it reached 0.5 McFarland standard turbidity, and then 100 μl of bacterial culture was added to each well of a 96-well microtiter plate containing sub-inhibitory concentrations (SIC) of plant extracts. After incubation at 37 °C for 24 h, each microtiter plate well was washed twice with phosphate buffer saline (PBS) and dried overnight. After that, the wells were stained with 0.1% crystal violet solution (Adwic – El Nasr Pharmaceutical Co. Cairo, Egypt) for 20 min at room temperature (28 ± 2 °C). Subsequently, the plate was washed with water and air-dried. Stained biofilms were dissolved in 200 μl of 95% (v/v) ethanol for about 30 min. Finally, the absorbance was taken at 595 nm. The percentage of biofilm formation inhibition was calculated using the following Eq. 1:

1 $${\text{Biofilm inhibition percentage}} = \left[ {\left( {{\text{OD Growth Control}}{-}{\text{OD Experimental}}} \right)/{\text{OD Growth Control}}} \right] \times 100.$$

### Synergistic effects of amoxicillin-clavulanic and selected plant extracts on the selected strain using L-optimal mixture design

L-optimal mixture design was performed by using the Design Expert Software (Version 11 Stat. Ease Inc., Minneapolis, USA) to study the effect of the interaction of the ethanolic plant extract compounds (Chamomile, Sage, and Cinnamon) and Antibiotic to obtain the significant formulation toward the response of *L. monocytogenes* ATCC7646 biofilm inhibition. Table 1 shows that the total number of mixture formulations was 20 experimental trials. The desired responses for each test run were computed, and the optimal combinations were predicted using the L-optimal mixture design. Model results were statistically analyzed^20^ to analysis of the variance (ANOVA), with significance at *p* ≤ 0.05. The parameters used in evaluating and selecting the best-fitted model are *F*-test statistic, probable error value (*p-*value), coefficient of determination (R^2^), adjusted-R^2^, predicted-R^2^, mean, standard deviation, and adequate precision. The mathematical model of multiple regression analysis (the second-order polynomial and fitted to special cubic and quadratic models equation) for representing response expressed as the biofilm inhibition percentage against planktonic cells of *L. monocytogenes* was calculated according to the following Eqs. 2 and 3:

2 $$Y=C0+ \sum_{{\text{i}}=0}^{{\text{n}}}{\text{CiXi}}+\sum_{1\le {\text{i}}<{\text{j}}}^{{\text{n}}}{\text{CijXiXj}}$$

3 $$Y=C0+ \sum_{{\text{i}}=0}^{{\text{n}}}{\text{CiXi}}+\sum_{1\le {\text{i}}<{\text{j}}}^{{\text{n}}}{\text{CijXiXj}}+\sum_{1\le {\text{i}}<{\text{j}}<\mathrm{k }}^{{\text{n}}}{\text{CijXiXjXk}}$$

where Y = the predicted response (dependent variable); C0, Ci, and Cij = the constant, linear, and interactive coefficients, respectively; Xi, Xj, and Xk = independent variables; and n = the number of variables.

**Table 1:** L-optimal mixture design matrix, the results of actual values, analysis of variance (ANOVA), and fit statistics of variables affecting antibiofilm activity by ethanolic plant extracts mixture against *L. monocytogenes* ATCC7646.

Run (No.)	Components concentration (mg/ml)			% Inhibition								
	CH	SV	CN	AB	Actual value	Predicted value						
1	0.12	0.15	0.19	0.05	89.16	89.17						
2	0.16	0.13	0.11	0.1	91.02	89.7						
3	0.14	0.11	0.15	0.1	90.40	91.11						
4	0.06	0.15	0.19	0.1	89.78	89.7						
5	0.12	0.15	0.14	0.09	85.44	85.67						
6	0.12	0.09	0.19	0.1	90.09	89.9						
7	0.18	0.09	0.13	0.1	91.64	91.76						
8	0.18	0.09	0.19	0.05	89.78	89.77						
9	0.15	0.14	0.16	0.05	93.49	93.23						
10	0.18	0.15	0.07	0.1	91.02	91.37						
11	0.14	0.11	0.19	0.06	93.49	93.51						
12	0.18	0.09	0.19	0.05	89.78	89.77						
13	0.18	0.03	0.19	0.1	88.85	88.76						
14	0.18	0.15	0.18	0	94.42	94.47						
15	0.18	0.15	0.12	0.05	94.11	94.12						
16	0.18	0.09	0.13	0.1	91.64	91.76						
17	0.12	0.15	0.19	0.05	89.16	89.17						
18	0.16	0.07	0.18	0.09	93.49	93.55						
19	0.18	0.15	0.12	0.05	94.11	94.12						
20	0.12	0.15	0.14	0.09	85.44	85.67						

Source	Model	^(1)^Linear mixture	AB	AC	AD	BC	BD	CD	ABC	ABD	ACD	BCD
Statistical analysis of variation (ANOVA)												
* df*	13	3	1	1	1	1	1	1	1	1	1	1
* F-*Value	22.1	30.9	47.5	2.4	3.9	12.1	12.4	12.6	18.8	29.2	0.005	7.3
* p-*Value	0.001*	0.001*	0.001*	0.171	0.093	0.013*	0.013*	0.012*	0.005*	0.002*	0.94	0.036*
Std. Dev	0.66		Adjusted R^2^	0.94								
Mean	90.82		Predicted R^2^	0.88								
R^2^	0.98			Adeq.precision		15.89						

No., number; CH, Chamomile; SV, Sage; CN, Cinnamon and AB, Antibiotic. p, corresponding significance level; F, corresponding significance level; SD, Standard Deviation; R^2^, Determination coefficient; Adj., Adjusted; Pred., Predicted; Adeq., Adequate. *Significant at 0.05 level.

Contour plots and three-dimensional response surface graphs allow visualization of responses under different parameters by connecting all points within two-dimensional and three-dimensional, respectively, to ease the interpretation of the model results. The plots also help visualize results when varying the constraints from 0 to 1 to obtain precise optimum points, which were tested experimentally.

### Gas Chromatography and Mass spectroscopy analysis (GC/MS) analysis of ethanolic plant extracts mixture

The mixture of ethanolic plant extracts was analyzed using GC/MS (Shimadzu GCMS-QP2020, Tokyo, Japan) at the Center for Drug Discovery Research and Development Faculty of Pharmacy, Ain Shams University, Cairo, Egypt. Column: Rtx-1MS fused bonded column (30 mx 0.25 mm i.d. × 0.25 µm film thickness, Restek, USA). Ionization mode: ionization voltage, 70 eV; ion source, 200 °C. Temperature program: 45 °C (2 min)–300 °C (5 min) and kept constant at 300 °C for 5 min (isothermal). The injector temperature is 250 °C. The carrier gas is helium, and the flow rate is 1.41 ml/min. Diluted samples: (1% v/v). The sample was injected with split mode (split ratio 1:15). Searcehed library: Wiley & Nist Mass Spectral Data Base library.

### Cytotoxicity of ethanolic plant extracts mixture on Vero cell line

The cytotoxic effect of the ethanolic plant extracts mixture was tested on the Vero cell line (a normal kidney CCL-81) (American type culture collection (ATCC)) using the MTT (3-(4,5-dimethylthiazol-2-yl)-2-5-diphenyltetrazolium bromide) (Bio Basic Inc., Toronto, Canada) assay^21^. This experiment was conducted at the Science Way for Scientific Research and Consultations Company in Cairo, Egypt. The 96-well tissue culture plate was inoculated with 100 µl/ well of standard cell viability (1 × 10^5^ cells/ml) and incubated at 37 °C for 24 h to form a complete monolayer sheet. After the confluent sheet of cells was formed, the growth medium was decanted from 96-well microtiter plates, and the cell monolayer was washed twice with wash media. Two-fold dilutions of the mixture of ethanolic plant extracts were made in an RPMI medium with 2% serum (maintenance medium). In each well, 0.1 ml of each dilution was tested, with three wells serving as controls and receiving only maintenance medium. The plate was incubated at 37 °C and examined. MTT solution was prepared (5 mg/ml in PBS) (BIO BASIC CANADA INC). Twenty microliters of MTT solution were added to each well and placed on a shaking table at 150 rpm for 5 min to mix the MTT into the media. The cells were then incubated at 37 °C, 5% CO_2_ for 4 h to allow the MTT to be metabolized. Resuspend formazan (MTT metabolic product) in 200 µl DMSO and shake at 150 rpm for 5 min on a shaking table to mix the formazan into the solvent. In a microtitre plate reader, the absorbance for each well was measured at 560 nm, and the optical density was expressed as the cell quantity. Morphological cells from a cytotoxicity study were examined for any physical signs of toxicity after being treated with an ethanolic plant extract mixture. The changes include partial or complete monolayer loss, rounding, shrinkage, or cell granulation.

### Application of plant extracts mixture against biofilm formation on stainless steel milk tank surface during white soft cheese manufacture

Fresh buffalo's milk was obtained from the farm of Faculty of Agriculture, Cairo University, on the experiment day. Chemical parameters of the used milk were determined using a milk analyzer (LCD display-4 lines × 16 characters, 100-240V-1.6A max., Bulgaria); Microbial rennet (Reniplus NG) was provided by Caglio Star, Murcia, Spain, corresponding to a thermolabile enzyme obtained by *Mucor miehei* fermentation. Calcium chloride anhydrous (C1016) was obtained from Sigma-Aldrich Company. Food-grade fine salt was obtained from El-Nasr Salines Company, Egypt.

#### *L. monocytogenes* ATCC7646 biofilm formation on stainless steel containers

Stainless steel containers were obtained from local markets and selected to resemble the material and design of bulk milk tanks in dairy plants as much as possible. *L. monocytogenes* ATCC7646 biofilm was formed in this container^22^. After confirmation of biofilm formation, the cups were thoroughly washed, air-dried, and ready for the second step.

#### Incidence of detached *L. monocytogenes* ATCC7646 organism from biofilm in the stainless steel containers into milk during storage

The fresh raw buffalo's milk was laboratory pasteurized by heating for 30 min at 62.8 °C, then immediately cooled in ice to simulate batch pasteurization^23^. Laboratory pasteurized milk (LPM) was then added to the previously prepared stainless steel cups with *L. monocytogenes* ATCC7646 formed biofilm in 3 groups; a control group without any additives, a second group at which LPM was inoculated with ethanolic sage extract (0.29 mg/ml) after cooling, and the last group was also inoculated with the ethanolic extracts mixture containing Chamomile 0.18, Sage 0.15 and Cinnamon 0.18 mg/ml. These cups for the 3 groups were duplicated to be stored as one part at the refrigerator temperature (4–6 °C) and the other part at room temperature (20–25 °C) for 12 h. Milk samples were taken (immediately after preparation, after 6 h, and after 12 h of storage), and the count of *L. monocytogenes* was determined using the standard plate count method on a specific medium according to ISO 11,290–1:2017 protocol^24^. The average of three trials was calculated.

#### Studying the antibacterial effect of the ethanolic Sage extract and the mixture of the selected extracts on L. monocytogenes ATCC7646 survival in white soft cheese during storage

White soft cheese was prepared using the procedure described by Abdel-Salam and Saad^25^ from 3 milk groups previously explained and then stored in the refrigerator (to simulate white soft cheese manufacture on a large scale). LPM samples were reheated at 37 °C for adding Calcium chloride, Sodium chloride, and rennet at the ratio of 0.02%, 3.00%, and 0.10 g/l, respectively, according to the manufacturer's instructions. Milk samples were then stirred well and left for about 4 h in the same stainless steel cups at 42 °C till coagulum formation, and then the curd was scooped into a gauze-covered strainer for whey drainage. The cheese block was then divided into suitable pieces, covered with drained whey of each group, and tightly closed in plastic containers. Cheese containers were stored in a refrigerator (6 ± 1 °C) for 14 days. During storage, *L. monocytogenes* ATCC7646 was enumerated in cheese samples (for the 3 groups) day after day. The experiment was done in triplicate, and average results were recorded.

#### Sensory evaluation of white soft cheese

The impact of fortifying cheese with ethanolic Sage extract and the mixture of the selected extracts on the sensory properties of white soft cheese as compared with control one was assessed according to the method cited by Ahmed, et al.^26^. Laboratory-manufactured cheese curd was examined for its physical parameters immediately after manufacture as zero time and on intervals during storage (6 °C/14 days). A panel test was done by 5 expert panelists (for each sample) from Food Hygiene and Control Department staff members, Faculty of Veterinary Medicine, Cairo University. Samples were evaluated for flavour (40 points), body and texture (40 points), colour and appearance (10 points), salt (5 points), and style (5 points).

### Statistical analysis

Data were analyzed using IBM ® SPSS ® statistics software version 19 based on Duncan'sMultiple Range Tests at 0.05^27^. The significance of differences between groups was determined through a one-way analysis of variance (ANOVA).

### Statement

Samples of plants were collected with the consent of the Faculty of Agriculture. The authors guarantee that all procedures were carried out in compliance with the rules and regulations that applied. The complete experimental protocol was approved by the Ethics Committee of The Faculty of Agriculture, Ain Shams University with approval No. (2023-11-03).

## Results and discussion

### Evaluation of the antibacterial activity of medicinal plant extracts

The antibacterial activity of the four ethanolic extracted medicinal plants was assessed against four foodborne bacterial strains belonging to three strains of Gr^+ve^ bacteria (*S. aureus* ATCC5638, *B. cereus* ATCC11778, and *L. monocytogenes* ATCC7646) and one strain of Gr^−ve^ bacteria (*S. Typhimurium* ATCC25566) using disc diffusion technique. According to the data in Table 2, ethanolic plant extracts significantly impacted foodborne bacterial strains. All the tested ethanolic plant extracts and Antibiotic (control sample) had a high significance (*p* < 0.05) against *L. monocytogenes* ATCC7646 with 21.8 mm of a mean inhibition zone diameter, followed by *S. Typhimurium* ATCC25566 or *S. aureus* ATCC5638 with 20.3 mm mean inhibition zone diameter. In contrast, the least significant effect of ethanolic plant extracts (17.4 mm mean inhibition zone diameter) was obtained against *B. cereus* ATCC11778. When compared to the tested Antibiotic (25.0 mm a mean inhibition zone diameter), the ethanolic plant extract of Sage (24.1 mm a mean inhibition zone diameter) was the most significant (*p* < 0.05) and effective in inhibiting all tested foodborne bacterial strains, followed by Cinnamon extract with 18.1 mm of a mean inhibition zone diameter. Chamomile and Marigold suppressed pathogenic bacterial strains with mean inhibition zone diameters of 16.2 mm and 16.3 mm, respectively. As shown by the ANOVA test, results in Table 2 show an increase in *F*-values, suggesting a lower *p* value for each model, intercept, pathogenic strains, and ethanolic plant extracts. The data also demonstrated a strong R^2^ between plant extracts and suppressed pathogenic strains (0.85), indicating that the model described 85% of the overall variation.

**Table 2:** Medicinal plants extracted by ethanol against foodborne pathogens.

Pathogenic bacteria	Inhibition zone diameter of ethanolic plant extracts (mm)					Mean
	CH	CL	SV	CN	AB	
*Listeria monocytogenes* ATCC7646	18.5^aD^	19.5^aD^	24.5^bB^	21.5^aC^	25.3^bA^	21.8^a^
*Salmonella Typhimurium* ATCC25566	15.5^cD^	16.3^cC^	26.0^aA^	17.5^bB^	26.5^aA^	20.3^b^
*Staphylococcus aureus* ATCC5638	16.5^bD^	17.5^bC^	22.5^ dB^	21.5^aB^	23.5^cA^	20.3^b^
*Bacillus cereus* ATCC11778	14.5^dC^	12.0^dD^	23.5^cB^	12.25^cD^	25.0^bA^	17.4^c^
Mean		16.2^C^	16.3^C^	24.1^A^	18.1^B^	25.0^A^

Source	*df*	*F*-value	*p* value	R^2^		
Statistical analysis of variance (ANOVA)						
Corrected model	7	43.1	0.0001*	0.85		
Intercept	1	6987.0				
Pathogenic strains	3	14.9				
Plant extracts	4	64.2				

CH, Chamomile; CL, Marigold; SV, Sage; CN, Cinnamon; Ab, Antibiotic; df, degree of freedom; *p*, corresponding significance level; *F*, the corresponding level of significance; R^2^, Determination coefficient and *, Significant. ^a, b^ Values with small letters in the same column having different superscripts are the significant difference (at *p* ≤ 0.05) between different pathogenic bacteria and the same plant extract. ^A, B^ Values with capital letters in the same row having different superscripts are the significant difference (at *p* ≤ 0.05) between various plant extracts and the same pathogenic bacteria.

From the above results, it could be observed that the ethanolic extracts of the tested plants might be a more effective antibacterial agent than an antibiotic, and they could be used as antimicrobial agents on a commercial scale. The methanol and ethanol extracts of the grape, mulberry, mallow, and lemon leaves for their antibacterial activity against *S. aureus*, *E. coli*, *P. aeruginosa*, and *Salmonella* sp^28^. They revealed that ethanol extracts from these plants had stronger antibacterial action than methanol because this solvent might dissolve polar and nonpolar molecules, besides a broad spectrum of plant-derived chemicals. On the other hand, ethanol had lower toxicity than methanol. Furthermore, the ethanol extracts of *Punica granatum*, *Syzygium aromaticum*, *Zingiber officinales*, and *Thymus vulgaris* were highly effective with varying efficiency against *B. cereus*, *S. aureus*, *E. coli*, *P. aeruginosa*, and *S. Typhi,* respectively, while *Cuminum cyminum* extract was only effective against *S. aureus*^29^. The most significant plant extracts were those from *P. granatum* and *S. aromaticum*. *Salvia officinalis* and *Psidium guajava* extracts significantly inhibit the growth of *S. aureus*, whereas *Olea europaea* and *Morus alba* extracts exhibit antibacterial activity versus *B. cereus*. They added that *O. europaea* and *S. officinalis* extracts could prevent *E. coli* and *S. entritidis* from growing^30^.

### Determination of MIC and MBC

**Figure 1:**
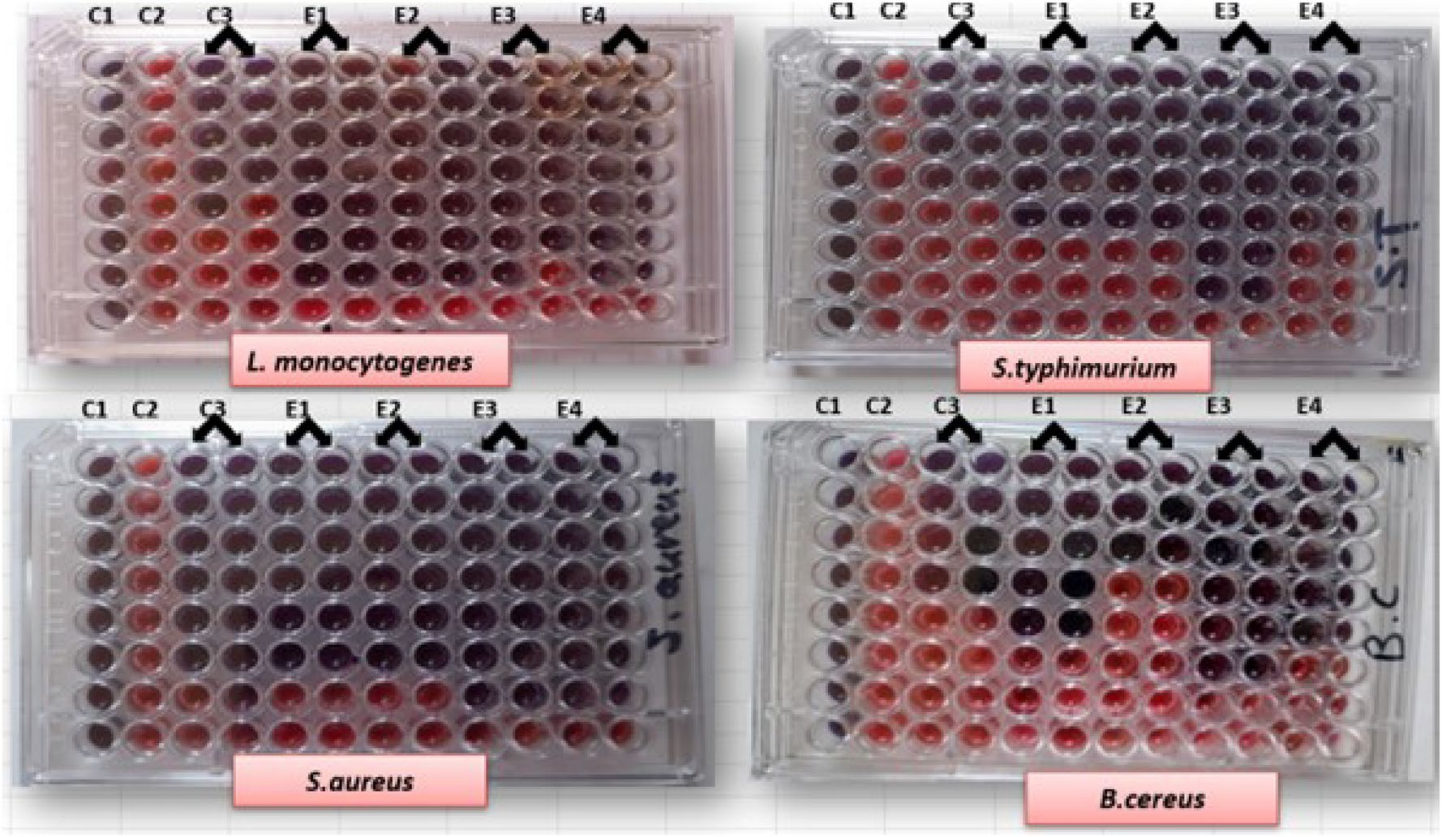
Minimum inhibitory concentration (MIC) of selected pathogenic bacteria by different ethanolic plant extracts (mg/ml). Column C1, sterility control (borth + indicator), no bacterial suspension and replaced by 10 μl of nutrient broth; column C2, control without plant extract (bacteria + broth + indicator) and two columns C3 positive control (Antibiotic in serial dilution + broth + indicator + bacteria). Columns ranged from E1–E4 ethanolic plant extract of tested plants, Chamomile, marigold, Sage and Cinnamon, respectively (in serial dilution in wells + broth + indicator + bacteria).

In this investigation, the MIC was determined using the microdilution broth susceptibility method, which resulted in a colour change from violet to pink, indicating that the tested ethanolic plant extracts (Chamomile, Marigold, Sage, and Cinnamon) inhibited the tested foodborne bacteria at concentrations ranging from 0.37 to 0.74 mg/ml, 0.62 to 2.50 mg/ml, 0.14 to 0.29 mg/ml and 0.39 to 1.60 mg/ml, respectively as compared with tested Antibiotic ranged from 0.04 to 0.39 mg/ml (Fig. 1 and Table 3). The volatile nature of the chemical components of diverse plant extracts may contribute to the difference in MIC of different plant extracts^29^. Data in Table 3 indicated that Chamomile, Marigold, Sage, and Cinnamon ethanolic extracts had MIC values of 0.37, 0.62, 0.29, and 0.39 mg/ml against *L. monocytogenes* ATCC7646; 0.74, 1.25, 0.14, and 1.60 mg/ml against *S. Typhimurium* ATCC25566; 0.74, 0.62, 0.14, and 0.39 mg/ml against *S. aureus* ATCC5638 and 0.74, 2.50, 0.29, and 0.78 mg/ml against *B. cereus* ATCC11778*,* respectively. The MIC value of positive control (Antibiotic) was 0.19, 0.39, 0.04, and 0.19 mg/ml against *L. monocytogenes, S. Typhimurium* ATCC25566*, S. aureus* ATCC5638 and *B. cereus* ATCC11778, respectively. Moreover, the MIC values of ethanolic extracts of grape and mulberry leaves against *P. aeruginosa* Ps9 varied from 0.08 to 0.16 mg/ml and against *S. aureus* St3, *E. coli* Ec3, and *S. Typhi* Sa1 were each 0.32 mg/ml^28^. For *Klebsiella* spp., the MIC value of ethanolic extracts of *Matricaria recutita* and *Moringa oleifera* ranged from 15.6 to 62.5 mg/ml, while for *S. aureus* and *E. coli*, it ranged from 7.8 to 62.5 mg/ml. Furthermore, *Piper betle* L. (*litlit*, *ikmo*) inhibited the growth of three test cultures, namely *P. aeruginosa* (MIC = 4.69 mg/ml), *S. aureus* (4.69 mg/ml), and *Candida albicans* (37.50 mg/ml)^31^. On the other hand, *Proteus mirabilis and P. aeruginosa* had lower MIC values, ranging from 7.8 to 31.25 mg/ml and 15.6 to 31.25 mg/ml, respectively^32^.The MBC of the plant extracts (Chamomile, Marigold, Sage, and Cinnamon) was studied to evaluate their bacteriostatic and bactericidal action. The dearth of bacterial growth of the tested strains'-streaked form changed colour well (indicating inhibitory activity) following their lowest MIC provided (Fig. 1) as evidence that the MBC occurred. The MBC values of the tested ethanolic plant extracts for the pathogenic bacterial strains ranged from 0.29 to 12.50 mg/ml (Table 3). The MBC values of all tested plant extracts were higher than that obtained by Amoxicillin-clavulanic Antibiotic against all tested strains, while the vice versa was valid for *S. Typhimurium* ATCC25566, which was more affected by Sage ethanolic extract than Antibiotic. Chamomile, Marigold, and Sage extracts had MBC/MIC ratios ranging from 1 to 2 against *S. aureus* ATCC5638 and *S. Typhimurium* ATCC25566. The Sage extract also exhibited bactericidal activity against *L. monocytogenes* ATCC7646, whereas Cinnamon extract and Amoxicillin-clavulanic Antibiotic resulting a bactericidal effect against all tested strains except *S. Typhimurium* ATCC25566. Both Chamomile and Marigold extracts recorded the bacteriostatic effect on the growth of *B. cereus* ATCC11778 and *L. monocytogenes* ATCC7646 at MBC/MIC ratio of 4:8, whereas the sage extract gave the same effect for the first strain only.

**Table 3:** Bactericidal and bacteriostatic effects of ethanolic plant extracts.

Ethanol plant extract	Bacterial pathogenic strains	MIC (mg/ml)	MBC (mg/ml)	Ratio MBC/MIC	Effect
Chamomile	*Staphylococcus aureus* ATCC5638	0.74	0.74	1	+
	*Bacillus cereus* ATCC11778	0.74	2.96	4	−
	*Listeria monocytogenes* ATCC7646	0.37	1.48	4	−
	*Salmonella Typhimurium* ATCC25566	0.74	1.48	2	+
Marigold	*Staphylococcus aureus* ATCC5638	0.62	1.25	2	+
	*Bacillus cereus* ATCC11778	2.50	10.00	4	−
	*Listeria monocytogenes* ATCC7646	0.62	5.00	8	−
	*Salmonella Typhimurium* ATCC25566	1.25	1.25	1	+
Sage	*Staphylococcus aureus* ATCC5638	0.14	0.29	2	+
	*Bacillus cereus* ATCC11778	0.29	1.16	4	−
	*Listeria monocytogenes* ATCC7646	0.29	0.58	2	+
	*Salmonella Typhimurium* ATCC25566	0.14	0.14	1	+
Cinnamon	*Staphylococcus aureus* ATCC5638	0.39	0.39	1	+
	*Bacillus cereus* ATCC11778	0.78	1.56	2	+
	*Listeria monocytogenes* ATCC7646	0.39	0.78	2	+
	*Salmonella Typhimurium* ATCC25566	1.60	12.5	8	−
Amoxicillin-clavulanic Antibiotic	*Staphylococcus aureus* ATCC5638	0.04	0.09	2	+
	*Bacillus cereus* ATCC11778	0.19	0.38	2	+
	*Listeria monocytogenes* ATCC7646	0.19	0.38	2	+
	*Salmonella Typhimurium* ATCC25566	0.39	1.56	4	−

MIC, minimum inhibitory concentration; MBC, minimum bactericidal concentration; Bactericidal ( +) =  ≤ 2, Bacteriostatic (−) =  ≥ 4.

From the aforementioned results it could be concluded that the growth of *L. monocytogenes* ATCC7646 was more sensitive for all tested ethanolic extracts, which recorded bactericidal effect by Sage and Cinnamon ethanolic extracts and bacteriostatic effect by Chamomile and Marigold. The MBC values of ethanolic extracts of grape and mulberry leaves ranged from 0.32 to 1.28 mg/ml^28^. All of the chosen strains were susceptible to the bactericidal effects of grape leaf extract at a ratio of 2, except for *S. Typhi* Sa1, which was susceptible to a bacteriostatic effect at a ratio of 4. As opposed to this, the mulberry extract exhibited a bacteriostatic impact on *P. aeruginosa* Ps9 and *S. Typhi* Sa1 with ratios of 4 and 16, respectively, and a bactericidal effect on *S. aureus* St3 and *E. coli* Ec3 with a ratio of 2. *P. granatum* and *S. aromaticum* extracts had MBC values of 5 mg/ml against *S. aureus* and 10 and 12.5 mg/ml against *P. aeruginosa,* respectively. Both of the extracts under examination were bactericidal^29^. Ethanolic *M. recutita* and *M. oleifera* extracts had the same bacteriostatic efficacy against *S. aureus* and *E. coli* in the 15.6 mg/ml to 125 mg/ml range. The MBC of the extracts ranged from 31.25 mg/ml to 125 mg/ml against *Klebsiella* spp., 15.6 to 62.5 mg/ml against *Proteus mirabilis*, and 31.25 to 62.5 mg/ml against *P. aeruginosa*^32^.

### Evaluation of the antibiofilm activity of medicinal plant extracts

The percentage of biofilm inhibition values above 50% is regarded as good, while between 0 and 49% is considered poor^33^. So, data presented in Table 4 and Fig. S1 show the highly significant inhibition of biofilm adhesives towards planktonic cells of *L. monocytogenes* ATCC7646, *S. Typhimurium* ATCC25566, and *B. cereus* ATCC11778 which ranged from 75 to 90% (84.4% of means), 81 to 86% (83.2% of means), and 75 to 99% (83.4% of means), respectively. In contrast, all tested extracts noticed the lowest biofilm inhibition percentage against *S. aureus* ATCC5638. Also, Marigold extract demonstrated a low level of significant inhibition of biofilm adhesion, with a mean inhibition of 57.7%. It’s worth noting that the biofilm inhibition percentage of Sage ethanolic extract was found to be comparable or slightly bigger than Amoxicillin-clavulanic Antibiotic against *L. monocytogenes* ATCC7646 and *B. cereus* ATCC11778 which increased about 14% for second strain. Also, Chamomile ethanolic extract increased biofilm inhibition of *S. Typhimurium* ATCC25566 by about 2%, whereas Cinnamon extract recorded biofilm inhibition lower than that of an antibiotic by all tested extracts. Moreover, Sage obtained the highest biofilm inhibition percent of *L. monocytogenes* ATCC7646, followed by Cinnamon and Chamomile extracts being 90, 85, and 82 mean%, respectively. Also, it could be noticed that a good R^2^ between plant extracts and inhibited biofilm pathogenic strains (0.93), showed that the model explained 93% of the overall variation. So these extracts where chosen for the following experiment to detect the optimum mixture between them against planktonic cells of *L. monocytogenes* ATCC7646. Antibacterial agents' capacity to suppress the formation or breakdown of biofilms holds promise for minimizing microbial colonization of surfaces and epithelial mucosa^34^. The action of essential oils on the biofilm is comparable to what has been observed with antibiotics such as β-lactams. Bacteria growing in biofilms have been proven substantially more resistant to these antibiotics than planktonic bacteria. Adding essential oils reduced the metabolic activity of the *L. monocytogenes* biofilm. Exposure to essential oils is related to significant cell membrane damage and cell death^33^. The grape and mulberry leaf ethanolic extracts inhibited biofilm formation by 48–66% at SICs ranging from 0.04 to 0.16 mg/ml. Both extracts had remarkable biofilm inhibitory activity (57–66%) against *P. aeruginosa* Ps9, and *E. coli* Ec3. At a SIC of 0.16 mg/ml, the grape extract inhibited *S. Typhi* Sa1 by 51%, followed by the mulberry (48%)^28^. It might be attributed to the capacity of phenolic acids in both extracts to suppress fimbriae synthesis and reduce the extracellular polymeric material essential for biofilm formation^35^. All the tested Eugenia species inhibited *P. aeruginosa* adhesion by more than 50%, showing anti-attachment solid activity. Most plant extracts also inhibited *S. aureus* and *E. faecalis* adhesion^36^**.** The excellent ability of plant extracts to interfere with the initial stage of biofilm formation of the tested bacterial strains could be attributed to interference with forces (like Brownian, Lifshitz–Van der Waals, sedimentation, and electrostatic interaction) that promote bacteria deposition and adhesion to surfaces^37^. The plant extracts might further inhibit the availability of nutrients since a variety of organic and inorganic compounds and other nutrients are necessary for cell proliferation and, consequently, cell adhesion. The active plant extracts may reduce colonizing on various body surfaces and epithelial layers, reducing infections^36,38^. Furthermore, plant extracts could have interfered with any of the factors that cause resistance in biofilms, such as the presence of an extracellular polymeric matrix, which causes the strong attachment of microbes to surfaces and low antibiotic penetration, or increased activity of efflux pumps, which remove antimicrobial agents from cells. The plant extracts might have interfered with the bacteria's cell-to-cell communication mechanisms (quorum sensing), limiting biofilm growth^36,39^.

**Table 4:** Antibiofilm activity of the medicinal plant extracts according to sub-inhibitory concentration (at 1/2 MIC) against tested pathogenic strains.

Ethanol plant extracts	% Biofilm inhibition				Mean
	*Listeria monocytogenes* ATCC7646	*Salmonella Typhimurium* ATCC25566	*Staphylococcus aureus* ATCC5638	*Bacillus cereus* ATCC11778	
Chamomile	82^cA^	86^aA^	37^bD^	75^eC^	**70.0**^**ab**^
Marigold	75^ dB^	81^dA^	12^dC^	77^ dB^	**57.7**^**c**^
Sage	90^aAB^	83^bcC^	16^cD^	99^aA^	**72.0**^**a**^
Cinnamon	85^bA^	82^cdAB^	10^eC^	81^cAB^	**64.5**^**b**^
Antibiotic	90^aA^	84^bB^	41^aC^	85^bB^	**75.0**^**a**^
Mean	84.4^a^	83.2^b^	23.2^a^	83.4^b^	

Source	*df*	*F*-value	*p* value	R^2^	
Statistical analysis of variance (ANOVA)					
Corrected model	7	96.4	0.0001*	0.93	
Intercept	1	4422.7			
Pathogenic strains	3	213.0			
Treatment	4	8.9			

The numbers > 0 ≥ 50% show low activity; > 50% show high activity against the bacteria, according by Sandasi, et al. ^33^. df, degree of freedom; *p*, corresponding significance level; *F*, corresponding level of significance; R^2^, Determination coefficient and *, Significant. ^a, b^ Values with small letters in the same column having different superscripts are the significant difference (at *p* ≤ 0.05) between various plant extracts and the same pathogenic bacteria. ^A, B^ Values with capital letters in the same row having different superscripts are the significant difference (at *p* ≤ 0.05) between different pathogenic bacteria and the same plant extract.

### L-optimal mixture design of plant extracts' synergistic action against *L. monocytogenes* ATCC7646 and the efficiency of creating active formulations

**Figure 2:**
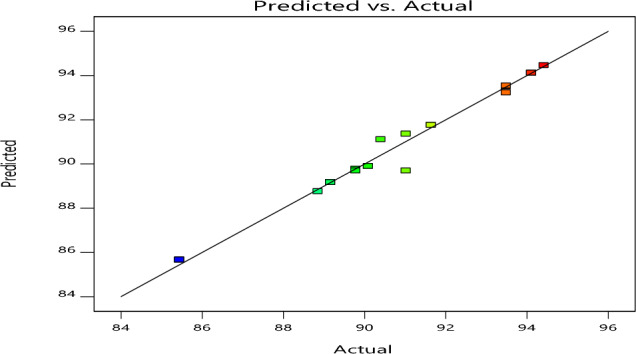
The actual and predicted values of L-optimal mixture design for inhibition of planktonic *Listeria monocytogenes* ATCC7646 cells.

Based on their 1/2 MIC values, the synergy between the three tested plant extracts (Chamomile, Sage, and Cinnamon) and Antibiotic as a positive control was estimated. To evaluate four mixture components and their experimental and predicted responses with each compound, the L-optimal mixture design was used, and a quadratic model was proved to be the best fit of all the models (linear, special cubic, and cubic). The predicted values obtained by model fitting are directly compared with the observed values shown in Table 1. In a 20-run trial, the value of inhibition of biofilm formation towards planktonic cells of *L. monocytogenes* ATCC7646 ranged from 85.44 to 94.42%, as shown in Table 1. The maximum inhibitory activity percentage (94.42% actual and 94.74% predicted value) was obtained in 14 runs with the following combination components (mg/l): Chamomile, 0.18; Sage, 0.15; and Cinnamon, 0.18. This run's component lacked antibiotics, indicating biofilm inhibition due to the use of only plant extracts, which resulted in a lower cost and bacterial multi-drug resistance. While the minimal inhibition percentage was 85.44, it was demonstrated in run numbers 5 and 20 with mixture components (mg/l) of Chamomile, 0.12; Sage, 0.15; Cinnamon, 0.14 and Antibiotic, 0.09. The statistical models and ANOVA analyses for active combination predicted against *L. monocytogenes* ATCC7646 biofilm are shown in Table 1. The unique cubic model had a highly significant effect with *F*-value of 22.14 and a *p* value of 0.001. All mixtures were highly significant (*F* value = varied from 7.3 to 30.9 and *p* value varied from 0.001 to 00), except for the synergistic mixture components Chamomile and Cinnamon (AC), Chamomile and Antibiotic (AD) and Chamomile, Cinnamon, and Antibiotic (ACD) with 2.4, 3.9, and 0.005 of *F*-value and 0.171, 0.093 and 0.94 of *p* value, respectively. The mean was 90.82, while the standard deviation was 0.66. The signal-to-noise ratio is a measure of adequate precision, and the ratio was 15.89, which was > 4; it was preferable and showed a good signal. The model's R^2^ coefficient had a high determination (0.98), indicating strong agreement between the experimental (R^2^ = 0.94) and predicted (R^2^ = 0.88) values. The polynomial regression model agreed with the experimental results (Fig. 2). The mathematical model of multiple regression analysis (the second-order polynomial equation) for representing response expressed as the biofilm inhibition percentage against planktonic cells of *L. monocytogenes* ATCC7646*.* The final Eq. 4 in terms of four actual independent components (A: Chamomile, B: Sage, C: Cinnamon, and D: Antibiotic) was:

4 $$\begin{aligned} {\text{Y}} & = {118.40}{\text{A}} - {927.11}{\text{B}} - {581.74}{\text{C}} - {881.49}{\text{D}} + {1665.05}{\text{AB}} \\ & \quad + {578.22}{\text{AC}} + {901.35}{\text{AD}} + {3514.44}{\text{BC}} + {4274.68}{\text{BD}} \\ & \quad + {3303.91}{\text{CD}} - {2206.00}{\text{ABC}} - {2965.82}{\text{ABD}} \\ & \quad + {81.78}{\text{ACD}} - {9361.76}{\text{BCD}} \\ \end{aligned}$$

**Figure 3:**
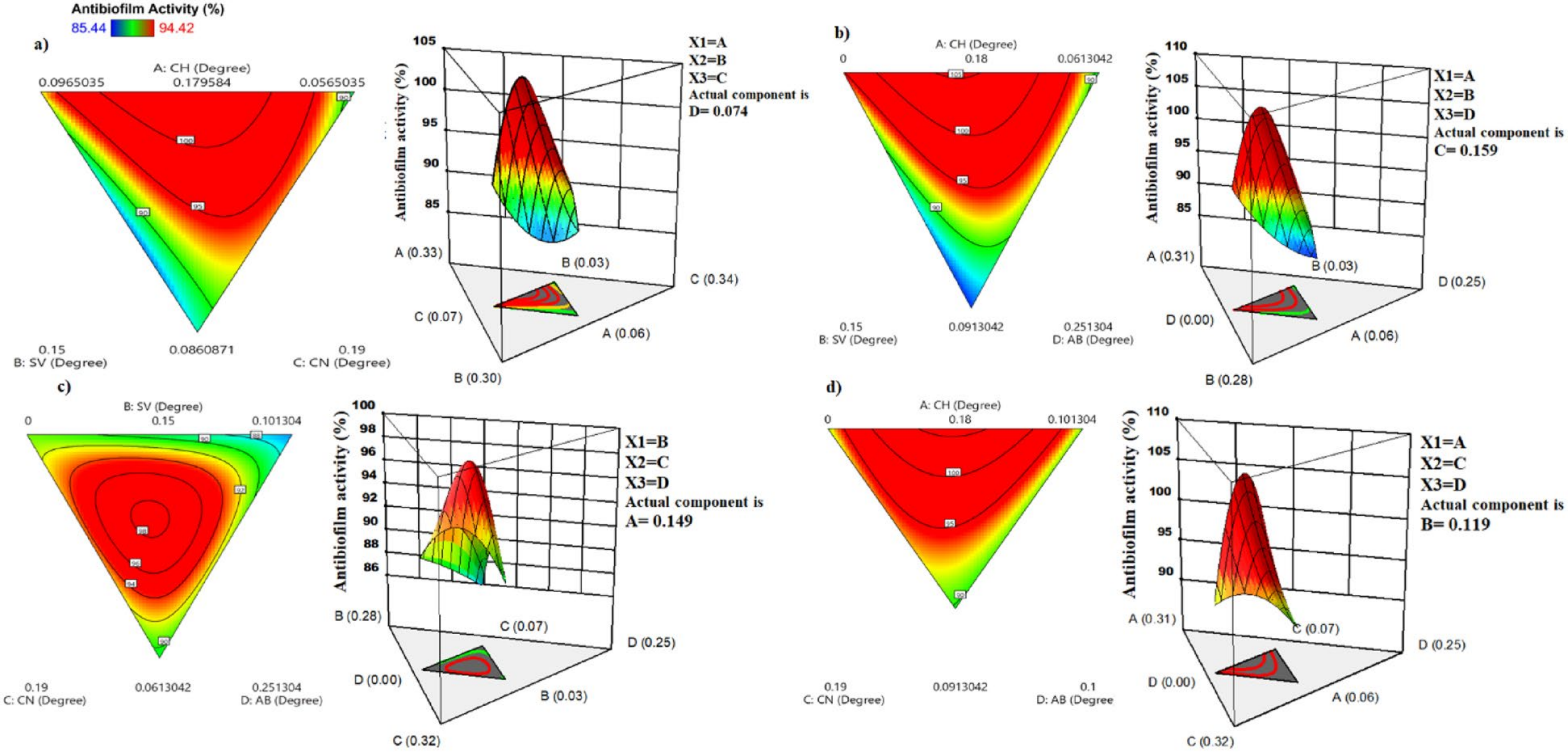
Contour plots and three-dimensional response surface and showing the antibiofilm effect of a mixture of Chamomile, Sage, and Cinnamon and an antibiotic against *Listeria monocytogenes*ATCC7646. (**a**) ethanolic mixture of Chamomile, Sage and Cinnamon, and the other independent antibiotic component was kept. (**b**) ethanolic mixture of Chamomile, Sage and Antibiotic, and the other independent component of Cinnamon was kept. (**c**) ethanolic mixture of Sage, Cinnamon and Antibiotic, and the other independent component of Chamomile, was kept. (**d**) ethanolic mixture of Chamomile, Cinnamon and Antibiotic, and the other independent component of Sage, was kept. CH: ethanolic Chamomile extract, SV: ethanolic Sage extract, CN: ethanolic Cinnamon extract, and AB: Antibiotic.

The one-component and interaction between two-component plots were also systematically evaluated in an L-optimal mixture design for the best biofilm inhibition evidenced by models in Supplementary Figs. S2&S3. The interaction between all two-component appeared in non-parallel lines except for two-component of AC was presented in parallel lines. Three-dimensional response surface (3D images) corresponding two-dimensional contour plots were graphically based on the model equation to explain the interaction among each independent three-component and determine each component at optimum level inhibition for *L. monocytogenes* ATCC7646 biofilm, have been illustrated in Fig. 3a–d. The region of red colour in 3D surface and contour plots indicated the highest antibiofilm activity, while the yellow to blue colour indicated the medium and lowest antibiofilm activity. Figure 3a exhibited the response surface of Y indicates a hilling or valley, meaning the interaction between components of A, B, and C (ABC) was significant (*p* value = 0.005) with a negative main effect (− 2206.00) and the other independent component of D was kept at 0.074 mg/ml for recoding strong antibiofilm inhibition. Results also indicated the increase Y could be achieved when the Chamomile (A) ranged from + 0.06 to + 0.33, Sage (B) ranged from + 0.03 to + 0.30, and Cinnamon (C) ranged from + 0.07 to + 0.34, respectively. Figure 3b shows the response surface of Y indicates a hilling or valley, meaning the interaction components of ABD were significant (*p *value = 0.002) with a negative main effect (− 2965.82), and the other independent component of C was kept at 0.159 mg/ml to achieve a high percentage of antibiofilm inhibition. So, the Y increased by the components ranged from + 0.06 to + 0.31 for Chamomile (A), from + 0.03 to + 0.28 for Sage (B), and from 0.00 to + 0.25 for Antibiotic (D), respectively. As well as, Fig. 3c demonstrated that regression of Y indicates a hilling or valley, meaning significant (*p* = 0.036) the interaction between components of B, C, and D was significant (*p* = 0.005) with a negative main effect (− 9361.76) and the other independent component of A was kept at 0.149 mg/ml to attend a high antibiofilm inhibition. The increase Y could be achieved when the Sage (B) ranged from + 0.03 to + 0.28, Cinnamon (C) ranged from + 0.07 to + 0.32, and Antibiotic (D) ranged from 0.00 to + 0.25, respectively. While in Fig. 3d the 3D image and contour plot indicated that the Y-like peak and no-significant (*p* = 0.94) interaction components of ACD, and it was the positive main effect (+ 81.78) when the other independent component of B at 0.119 mg/ml to achieve a maximum percentage antibiofilm inhibition. The Y increased when the Chamomile (A) ranged from + 0.06 to + 0.31, Cinnamon (C) ranged from + 0.07 to + 0.32, and Antibiotic (D) ranged from 0.00 to + 0.25, respectively. The *Cinnamomum verum* had a great antibacterial and antibiofilm effect against *L. monocytogenes* with MIC values of 0.100 mg/ml^40^. Cinnamon essential oils inhibited the initial cell attachment completely and inhibited 61% of preformed biofilms after 1 h incubation. Eugenol, Cinnamaldehyde, and β -Caryophyllene, when used individually, had an impact on the inhibition of surface attachment and subsequent biofilm formation against both *L. monocytogenes* MTCC657 (67.42% ± 2.6, 60.71% ± 3.0, and 28.63% ± 2.4) and *S. Typhimurium* MTCC3224 (59.61% ± 2.4, 52.4% ± 3.1, and19.27% ± 2.0)^41^. The inhibitory effect on surface attachment and subsequent biofilm formation by Cinnamaldehyde/Eugenol combine was found to be significantly *(p* < 0.05) greater against both *L. monocytogenes* MTCC657 (89.16% ± 3.2) and *S. Typhimurium* MTCC3224 (82.30% ± 3.4) when compared to inhibition effect by β-Caryophyllene/Cinnamaldehyde (*L. monocytogenes* MTCC657 of 64.83 ± 3.6% and *S. Typhimurium* MTCC3224 of 56.86 ± 2.2%) and β-caryophyllene/Eugenol (*L. monocytogenes* MTCC657 of 71.44% ± 2.1 and *S. Typhimurium* MTCC3224 of 63.12% ± 2.4). Moreover, a combination Cinnamaldehyde/Eugenol mixture was found to be more effective than reducing conducted biofilms against the tested bacterial pathogens (*L. monocytogenes* MTCC657 of 89.16% ± 3.2 and *S. Typhimurium* MTCC3224 of 70.61% ± 2.7). Also, the Sage extracts reduced biofilm formation only at high concentrations (512 μg/ml), and combinations of nisin and sage extracts can inhibit biofilm formation by *L. monocytogenes*^42^**.**

### Analysis of ethanolic plant extracts mixture using GC–MS

**Figure 4:**
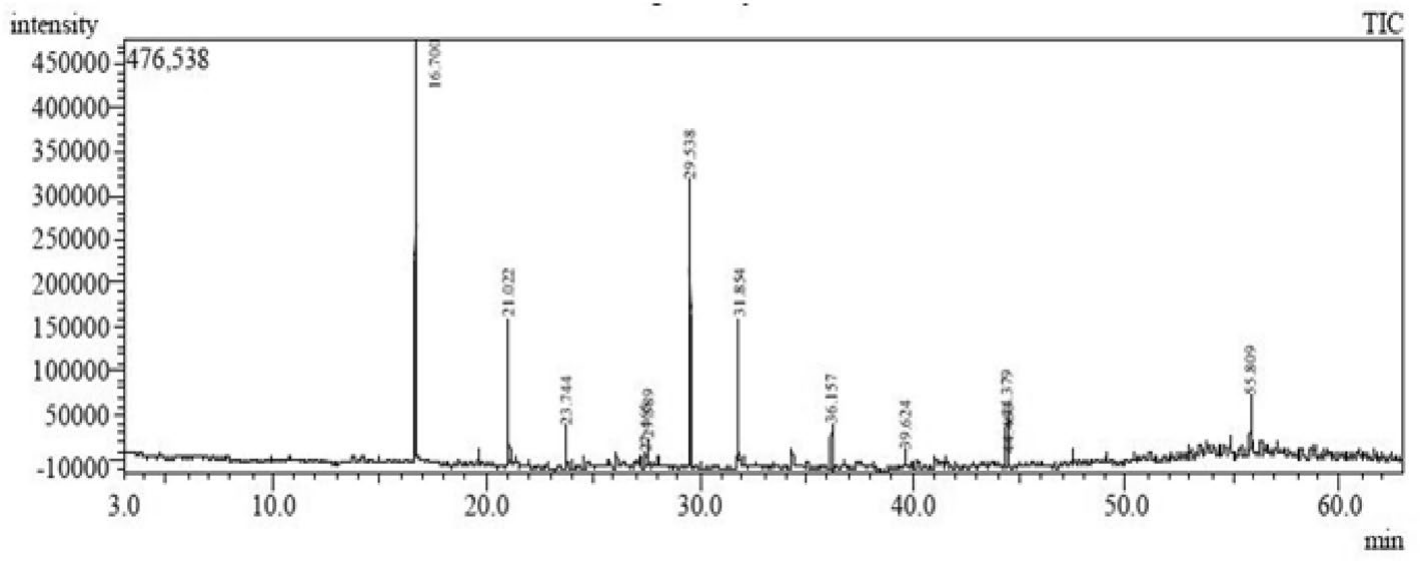
Chromatogram of Ethanolic plant extracts mixture by GC-Mass.

Data in Fig. 4 and Table 5 showed the phytochemical compounds of the ethanolic plant extracts mixture (Chamomile, Sage, and Cinnamon) resulting from GC/MS analysis, including their retention time (RT), peak area% (concentration), molecular weight, name of metabolite and mass spectra. The mixture is composed of 12 phytochemical compounds (peaks). On comparison of the mass spectra of the constituents with the NIST library, the twelve phytochemical compounds (2-Propenal, 3-phenyl-, Benzofuran, 2-Propenal, 3-(2-methoxyphenyl), Propane, 2-cyclohexyl-2-phenyl, Nerolidol isobutyrate, 2H-Pyran-3-ol, tetrahydro-2,2,6-trimethyl-6-(4-methyl-3-cyclohexen-1-yl)-, [3S-[3.alp, (Z)-2-(Hexa-2,4-diyn-1-ylidene)-1,6-dioxaspiro[4.4]non-3-ene, (4aR,5R,9aR)-1,1,4a,8-Tetramethyl-2,3,4,4a,5,6,7,9a-octahydro-1H-benzo[7]annulen-5-, Butyl citrate, Heptadecane, Phthalic acid, di(6-methylhept-2-yl) ester and 5-(7a-Isopropenyl-4,5-dimethyl-octahydroinden-4-yl)-3-methyl-penta-2,4-dien-1-ol) were characterized and identified (Table 5). The maximum area percentage was observed by 2-Propenal,3-phenyl- covered at RT of 16.7 min follew by 2H-Pyran-3-ol, tetrahydro-2,2,6-trimethyl-6-(4-methyl-3-cyclohexen-1-yl)-, [3S-[3.alp covered at RT of 29.538. This compound affects the cell membrane permeability, and confocal laser scanning microscopy images illustrate the detachment and killing of existing biofilms^43^. Dong, et al.^44^ added that it has anticancer, anti-inflammatory, and antioxidant properties. The minimum area % was recorded by Butyl citrate (0.76%) and Phthalic acid, di(6-methylhept-2-yl)ester (1.40%) covered at RT of 39.62 and 44.46 min, respectively. These compounds have antimicrobial, antioxidant, insecticidal, antineoplastic, and immunosuppressive effects^45^. Moreover, the first compound was used as a food additive, and the other compounds showed the same activities as antimicrobial, anti-inflammatory, anticancer, antioxidant, anti-tubercular, and anti-Alzheimer's^46^. Phenolic acids are important because of their pharmacological activities, such as antimicrobial, cytotoxicity, anti-inflammatory, and antitumor. In addition to these properties, the flavonoids act as powerful antioxidants, scavenging free radicals to protect the human body from dangerous diseases, and this property is dependent on the attachment and number of hydroxyl groups^8^. Polyphenolic extract has anti-swarming activity on biofilm formation of *Chromobacterium violaceum* 026, *E. coli* K-12 and *P. aeruginosa* PAO1 through targeting quorum sensing (QS) related violacein factors^47^. Recently, emerging evidence also indicated that natural products such as erianin (from Dendrobium chrysotoxum), isovitexin and parthenolide exhibited an inhibitory effect on cell adhesion, binding activity of fibronectin and QS factors, respectively, through targeting SrtA or downregulation of surface protein staphylococcal protein A (SpA) or blocking *P. aeruginosa* associated virulence factors, thereby impairing microbial biofilm formation^48^.

**Table 5:** GC/MS Analysis of the Ethanolic mixture plant extracts (Chamomile, Sage, and Cinnamon).

Peak	RT (min)	PA (%)	MW (mol)	Name of metabolite	Mass spectrum
1	16.7	34.82	132	2-Propenal, 3-phenyl-	
2	21.022	11.67	118	Benzofuran	
3	23.744	2.82	162	2-Propenal, 3-(2-methoxyphenyl)	
4	27.465	1.66	202	Propane, 2-cyclohexyl-2-phenyl	
5	27.589	1.86	292	Nerolidol isobutyrate	
6	29.538	22.46	238	2H-Pyran-3-ol, tetrahydro-2,2,6-trimethyl-6-(4-methyl-3-cyclohexen-1-yl)-, [3S-[3.alp	
7	31.854	10.10	200	(Z)-2-(Hexa-2,4-diyn-1-ylidene)-1,6-dioxaspiro[4.4]non-3-ene	
8	36.157	3.38	222	(4aR,5R,9aR)-1,1,4a,8-Tetramethyl-2,3,4,4a,5,6,7,9a-octahydro-1H-benzo[7]annulen-5-	
9	39.624	0.76	360	Butyl citrate	
10	44.379	3.46	240	Heptadecane	
11	44.46	1.40	390	Phthalic acid, di(6-methylhept-2-yl) ester	
12	55.809	5.61	288	5-(7a-Isopropenyl-4,5-dimethyl-octahydroinden-4-yl)-3-methyl-penta-2,4-dien-1-ol	

*RT, Retention Time; PA, Peak Area; MW, Molecular weight.

### Cytotoxicity of ethanolic plant extracts mixture on Vero cell line

**Figure 5:**
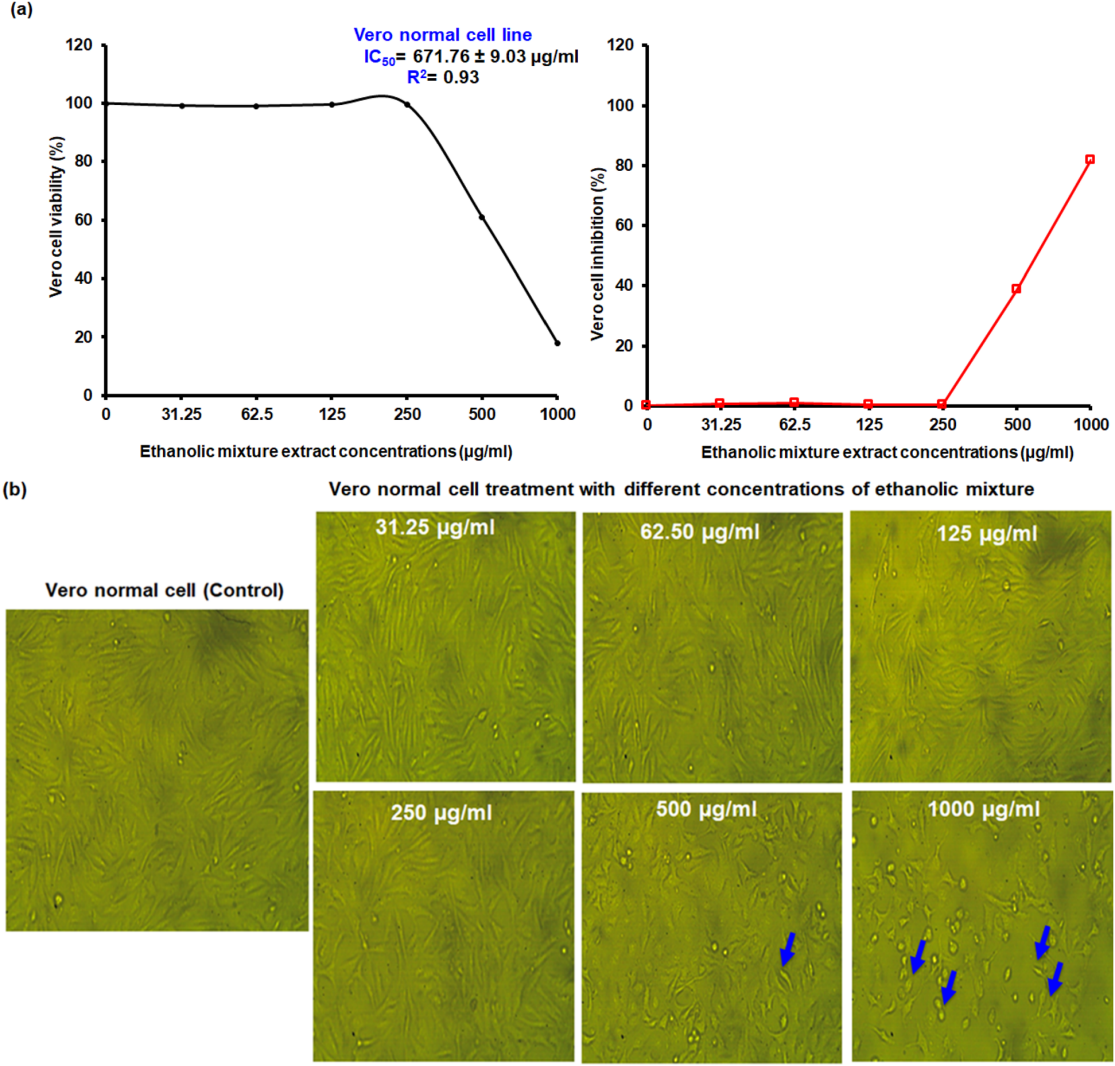
Vero normal cell line viability and inhibition percentage (**a**) and morphological changes of the cell line (**b**) after treatment with various concentrations of ethanolic mixture extract (ranged from 31.25 to 1000 µg/ml), photographed with an inverted phase-contrast microscope at a magnification of × 100. Apoptotic cells (cell shrinkage), cell debris, and major decreases in cell number are all indicated by the blue arrows on the image. An ethanol mixture extract included Chamomile, Sage, and Cinnamon extracts.

Vero is a normal kidney CCL-81 cell line. Cells are epithelial and adherent. This study used the MTT method to measure the cytotoxicity activity of an ethanol mixture (Chamomile, Sage, and Cinnamon) extract against the Vero cell line at six different concentrations. According to the results shown in Fig. 5 a, after being exposed to an ethanol mixture extract at a concentration of up to 250 µg/ml for 24 h, the Vero cell maintained a percentage of viable cells that ranged from 99.09 to 99.64%. Therefore, at 31.25, 62.50, 125, and 250 µg/ml concentrations, the ethanol mixture extract showed no cytotoxicity in the cell line. At a concentration of 500 µg/ml of ethanol mixture extract, the cell viability was reduced to 61.11% (with inhibition of 38.88%), but cytotoxicity was not observed because more than 50% of the cells remained alive. The cell viability was drastically reduced to 18.01%, and the toxicity increased to 81.99% when the extract of an ethanol mixture was used at a high concentration of 1000 µg/ml.

GraphPad Prism version 5 was used to determine the half-maximal (50%) inhibitory concentration (IC_50_). The IC_50_ values were calculated as 50% cell viability inhibition^28^. Vero cells had an IC_50_ value of 671.76 ± 9.03 µg /ml and a high R^2^ of 0.93 (Fig. 5a).

The morphological changes of the cell line were examined after treatment with various concentrations of ethanolic mixture extract (ranging from 31.25 to 1000 µg/ml), using an inverted phase-contrast microscope at a magnification of 100 × (Fig. 5 b). When treated with an ethanolic mixture extract of up to 250 µg/ml, the Vero cells survived and had normal adherent cells. While the cell morphology changed clearly, particularly at higher concentrations (1000 µg/ml) of ethanolic mixture extract, it appears apoptotic cells (cell shrinkage), cell debris, as well as major decreases in cell number indicate cell death compared to control. Five medicinal plants (*Acacia tortilis* (Hayne), *Fuerstia Africana* T.C.E. Friers, *Manilkara discolor* (Sond.) J.H.Hemsl., *Pentas lanceolata* (Forssk.) Defleurs, and *Sericocomopsis hildebrandtii* Schinz) used in Kenya and higher IC_50_ values greater than 0.100 mg/ml, meaning its non-cytotoxicity towards Vero cell lines^49^. The IC_50_ values for the methanolic aerial parts and roots of *F. africana* extract were > 500 µg/ml and 366.38 µg/ml, of *P. lanceolata* was > 500 µg/ml, and of *S. hildebrandtii* were > 500 µg/ml and 93.97 µg/ml, respectively. Whereas methanolic Aerial parts and roots *A. tortilis* and *M. discolor* had IC_50_ values of > 100 µg/ml. Furthermore, *Tieghemella heckelii* stem bark had an IC_50_ value greater than 0.01 mg/ml (ranging from 0.051 to 0.192 mg/ml) and had an 80.2% viability, neither of which indicated any apparent cytotoxicity towards the Vero cell line^50^. IC_50_ for wormwood ethanolic leaf extract against the normal kidney cell line was 500 µg/ml^51^. In vitro*,* the cytotoxic activity of the ethanolic leaf extract of *Eucalyptus camaldulensis* against normal human fibroblast cell line OUMS with IC_50_ value of 165.9 µg/ml ± 10.3^52^.

### Application of plant extracts mixture against biofilm formation on stainless steel milk tank surface during white soft cheese manufacture

*L. monocytogenes* has an excellent potential to form biofilms on materials such as stainless steel, rubber, and plastics. These materials are commonly set up in dairy instruments in all plants, milk handling equipment, milk lines, milk tanks, transportation trucks, or even sampling equipment, which may contribute to the ability of *L. monocytogenes* biofilms presence in dairy processing plants^53^. The incidence of *L. monocytogenes* biofilm in dairy plants was observed in several studies^54^. Prevention of the establishment of biofilms in milking equipment is a crucial step in fulfilling the requirement for safe and high-quality milk and dairy products^53^. On another aspect, the ability of *L. monocytogenes* cells attached to milk equipment surfaces to detach and multiply in dairy products constitutes a significant risk to the consumer, as the detached cells showed higher tolerance to stressful conditions than suspended cells^55^.

For the public health significance of *L. monocytogenes* biofilm existence in milk and dairy products plants and from the results mentioned above, another part of this study was directed to examine the incidence of *L. monocytogenes* ATCC7646 organism shedding from the formed biofilm in the stainless steel containers to milk during different storage conditions (time and temperature) and its persistence in soft cheese produced from contaminated milk concerning the possible inhibiting effect of the added ethanolic Sage extract and the selected extracts mixture. Data presented in Table 6 showed that *L. monocytogenes* ATCC7646 detached cell count was significantly increased after 12 h of storage at 4 °C in the control group than other treated groups, which indicates the antimicrobial effect of ethanolic Sage extract and the mixture of the tested extracts against *L. monocytogenes* ATCC7646 even in the refrigerator. The bactericidal effect of the tested ethanolic extracts mixture was better than that of the ethanolic Sage extract as it significantly lowered the count of *L. monocytogenes* ATCC7646 after 6 h of storage (3.12 Log CFU/ml). Meanwhile, the ethanolic Sage extract reduced the count significantly after 12 h of storage (2.86 Log CFU/ml) at 4 °C. In parallel, the same samples were stored at 20 °C to compensate for the average room temperature, compared with refrigerator (4 °C) raw milk storage until the cheese production process started. Detached *L. monocytogenes* ATCC7646 cells were significantly increased after 6 h of storage in both controls (4.03 Log CFU/ml) and ethanolic Sage (3.54 Log CFU/ml) milk groups than the fortified group with the mixture of the extracts (3.04 Log CFU/ml) which was significantly lower than the control group even after 12 h of storage. That indicates the strong, persistent antibacterial effect of the tested ethanolic extracts mixture against *L. monocytogenes* during storage for 12 h either inside the refrigerator (4 °C) or outside at room temperature (20 °C), which have been tabulated in Table 6. *L. monocytogenes* showed greater survival capacity during storage at 4 °C compared to 22 °C with more than 2.5 reductions^56^.

**Table 6:** Survival of *L. monocytogenes* ATCC7646 detached cells from produced biofilm in stainless steel containers in raw milk stored for 12 h at 4 and 20 °C (Log^10^ CFU/ml).

Time (h)	Storage at 4 °C			Storage at 20 °C				
	T1	T2	T3	T1	T2	T3		
0	4.04^Aa^	3.79^Ab^	3.94^Ab^	4.04^Aa^	3.79^Ab^	3.94^Ab^		
6	3.90^Aa^	3.55^Ab^	3.12^Aa^	4.03^Ca^	3.54^Bb^	3.04^Aa^		
12	3.48^Ba^	2.86^Aa^	2.58^Aa^	3.95^Ba^	2.74^Aa^	2.87^Aa^		

Source	df	*F*-value	*p* value	R^2^	*df*	*F*-value	*p* value	R^2^
Statistical analysis of variance (ANOVA)								
Corrected model	8	6.5	.000	0.74	8	22.0	.000	0.91
Intercept	1	2659.3	.000		1	8953.9	.000	
Time	2	16.8	.000		2	32.5	.000	
Treatment	2	6.7	.007		2	37.7	.000	
Time * Treatment	4	1.3	.309		4	9.0	.000	

T1 = Control, T2 = ethanolic Sage extract, T3 = ethanolic extracts mixture (including Chamomile, Sage, and Cinnamon extracts). ^a,b^ Values with small letters in the same column having different superscripts are significant differences (at *p* ≤ 0.05) between the same treatment during different hours. ^A,B^ Values with capital letters in the same row with different superscripts are significant differences (at *p* ≤ 0.05) between treatments at the same time.

*indicates the interaction between time and treatment.

Raw milk stored at 4 °C in the stainless steel container with *L. monocytogenes* ATCC7646 biofilm for 12 h was then used for white soft cheese production. Detached *L. monocytogenes* ATCC7646 cells were counted after cheese curd production (zero-day) and periodically day after day till 14 days of storage at 4 °C or till the disappearance of the microorganism, as presented in Table 7. Refrigeration temperatures during storage and ethylene production during the cheese ripening process favour the growth of *L. monocytogenes*^57^**.** That cleared the significant increase of *L. monocytogenes* count in the control sample after production and during storage till samples spoilage after 10 days. On the other hand, the count was significantly lowered by using the sage extract after 4 days of storage (2.73 Log CFU/g) and continued to significantly decrease at each examination interval till the complete disappearance of cheese samples after 10 days of storage. Best results were achieved by the use of selected extracts mixture as a fortification in cheese; *L. monocytogenes* ATCC7646 count was lowered in this group than in other groups significantly from the second day of storage (2.54 Log CFU/g) till complete disappearance after only 8 days of storage. This ensures the favourable bactericidal effect of ethanolic Sage extract and the tested ethanolic extracts mixture even in the presence of salt, ripening byproducts, and cold temperature in white soft cheese, which are the optimum conditions for the growth of *L. monocytogenes* ATCC7646 as indicated in the control samples. The Sage extract significantly increased the antimicrobial activity of the fresh cheese against *L. monocytogenes* (1.2 Log CFU/g)^58^. Although predictive microbiology models recounting the growth/death kinetics of *L. monocytogenes* in variable cheeses have been widely recognized, the variability in physicochemical characteristics and technological parameters between different cheese types affects the microbial behaviour of *L. monocytogenes*, especially type and level of lactic acid bacteria used as a starter culture^56^. *L. monocytogenes* strains organized in biofilm, in Gorgonzola cheese processing plants located in Italy^59^. Also, biofilm-forming *L. monocytogenes* strains isolated at different points in Brazilian cheese processing plants, including the cooling chamber (n = 16), floor of the pasteurization room (n = 8), floor of cooling chamber (n = 32), plastic crates (n = 8), platform of the cooling chamber (n = 7), surfaces of worker's gloves (n = 3), and brine (n = 5)^60^.

**Table 7:** Survival of *L. monocytogenes* ATCC7646 in white soft cheese produced from contaminated milk from production and during storage at 4 °C for 14 days.

Time (day)	Treatment			
	T1	T2	T3	
0	4.41^Cb^	3.92^Bd^	3.64^Ac^	
2	4.26^Bab^	2.88^Acd^	2.54^Abc^	
4	4.00^Ca^	2.73^Bc^	2.10^Ab^	
6	3.90^Bab^	2.38^Acd^	1.28^Ab^	
8	3.89^Bab^	1.44^Ab^	0.00^Aa^	
10	S	0.00^Aa^	0.00^Aa^	

Source	*df*	*F*-value	*p* value	R^2^
Statistical analysis of variance (ANOVA)				
Corrected model	16	37.2	.000	0.946
Intercept	1	1685.5		
Time	5	52.8		
Treatment	2	94.5		
Time * Treatment	9	5.3		

T1 = Control, T2 = Sage ethanolic extract, T3 = ethanolic extracts mixture (including Chamomile, Sage, and Cinnamon extracts), d = days, S = Spoilage. ^a,b^ Values with small letters in the same column having different superscripts are significant differences (at *p* < 0.05) between the same treatment during different days. ^A,B^ Values with capital letters in the same row with different superscripts are significant differences (at *p* < 0.05) between treatments at the same time.

The sensory observation of food is a complex process affected by various elements, including the dairy product's flavour, texture, and appearance^61^. Herbal extracts could enhance sensory qualities, prevent lipid oxidation, and lengthen the shelf life of soft cheeses^62^.

Therefore, the sensory quality of produced white soft cheese fortified with ethanolic Sage extract and the tested ethanolic extracts mixture was important to be evaluated immediately after curdling and during storage, at 4 °C for 14 days, as presented in Table 8. From the production point, the soft cheese samples fortified with ethanolic extracts mixture were the most preferable to the panelist, with a significantly higher overall acceptability with a mean value of 92.2 ± 2.7 than control and Sage extract fortified cheese and the only sample graded as excellent. This highest overall acceptability is attributed to the high flavour (37.6 ± 1.5) and texture (36.4 ± 1.8) scores, even than the control group.

**Table 8:** Sensory evaluation of white soft cheese samples fortified with (ethanolic Sage 0.03% w/v and tested mixture extracts) from production and during storage at 4°C for 14 days.

Treatments	Grade	Score	Time (day)					
			Zero time	3	5	7	10	14
Control cheese	Flavor	40	33.8 ± 3.8	32.4 ± 3.9	31 ± 4.8	28.6 ± 3.6	26.4 ± 3.7	25.6 ± 3.0
	Texture	40	35.4 ± 2.6	34.2 ± 3.1	33.2 ± 2.3	31.8 ± 2.7	30.8 ± 2.0	29.2 ± 2.0
	Color	10	8.4 ± 1.3	7.6 ± 1.5	6.4 ± 1.4	5.6 ± 1.3	4.6 ± 1.3	4.6 ± 1.3
	Salt	5	4 ± 0.7	3.8 ± 0.4	3.2 ± 0.4	3 ± 0.0	3 ± 0.0	3 ± 0.0
	Style	5	4 ± 0.0	4 ± 0.0	3.8 ± 0.4	3 ± 0.0	3 ± 0.0	3 ± 0.0
	Overall acceptability	100	85.6 ± 7.0^Ab^	82 ± 6.9^Aa^	77.8 ± 7.6^ABb^	72 ± 6.1^BCa^	67.8 ± 4.9^Cb^	65.6 ± 5.7^Cb^
	Grade		B	B	C	C	C	C
Cheese with Sage extract	Flavor	40	34 ± 2.2	34.6 ± 1.5	32.8 ± 1.8	31.2 ± 1.3	29.8 ± 1.1	29 ± 1.2
	Texture	40	33.6 ± 3.5	33 ± 2.8	31.6 ± 2.1	30.6 ± 1.5	29.6 ± 0.9	28.2 ± 1.1
	Color	10	7.8 ± 0.4	7.4 ± 0.9	7 ± 0.7	6.8 ± 0.4	6 ± 0.7	5.8 ± 0.4
	Salt	5	4 ± 0.0	4 ± 0.0	3.8 ± 0.4	3 ± 0.0	3 ± 0.0	3 ± 0.0
	Style	5	4.6 ± 0.5	4 ± 0.0	3.8 ± 0.4	3.6 ± 0.5	3 ± 0.0	3 ± 0.0
	Overall acceptability	100	84 ± 3.0^Ab^	82.6 ± 3.8^ABa^	79 ± 3.0^Bab^	75.2 ± 2.4^Ca^	71.4 ± 2.3^Db^	69 ± 2.3^Dab^
	Grade		B	B	C	C	C	C
Cheese with extracts mixture	Flavor	40	37.6 ± 1.5	36 ± 2.8	35 ± 3.2	33.8 ± 3.3	32.4 ± 3.4	30.6 ± 1.1
	Texture	40	36.4 ± 1.8	35.8 ± 2.3	35 ± 1.4	33.2 ± 2.7	32.2 ± 1.8	30.8 ± 2.3
	Color	10	9 ± 0.7	8.2 ± 0.4	8.2 ± 0.4	7.8 ± 0.4	7 ± 0.7	7 ± 0.7
	Salt	5	4.4 ± 0.5	4.4 ± 0.5	4 ± 0.0	3.4 ± 0.5	3 ± 0.0	3 ± 0.0
	Style	5	4.8 ± 0.4	4.8 ± 0.4	4 ± 0.7	3.8 ± 0.4	3.2 ± 0.4	3 ± 0.0
	Overall acceptability	100	92.2 ± 2.7^Aa^	89.2 ± 4.4^ABa^	86.2 ± 4.0^BCa^	82 ± 4.6^CDa^	77.8 ± 5.0^DEa^	74.4 ± 3.3^Ea^
	Grade		A	B	B	B	C	C

Overall acceptability Grades: Grade A (Excellent): > 90%, Grade B (Good): 80–90%, Grade C (Fair): 60–80% and Grade D (Poor): < 59%. The results in the Table are the mean of five panelists. ± , Standard deviation. ^A,B^ Values with capital letters in the same row with different superscripts are significant differences (at *p* < 0.05) between the same treatment during different days. ^a,b^ Values with small letters in the same column having different superscripts are significant differences (at *p* < 0.05) between treatments at the same time.

On the contrary, the ethanolic Sage extract cheese samples had the lowest scores for texture, colour, and overall acceptability (33.6 ± 3.5, 7.8 ± 0.4, and 84 ± 3.0, respectively). The most obvious change was the colour of the cheese and abnormal flavour, especially after taste. As anticipated, overall acceptability ratings dropped as storage times increased. Although that, the mixture of the extracts fortified cheese showed the most stable parameters with good overall acceptability till the 7th day of storage, while control and sage extract cheese were graded as fair since the 5th day of storage. By the end of the storage period (14 days), all cheese samples were fair to the panelist but with the most acceptability and significant highest score recorded for the mixture of the extracts fortified cheese samples; 30.6 ± 1.1, 30.8 ± 2.3, 7 ± 0.7, and 74.4 ± 3.3 for flavour, texture, colour, and overall acceptability, respectively.

Recently, several studies have been interested in studying the effect of different herbal extracts on soft cheese sensory parameters to be used as natural additives for preservation. UF-soft cheese containing essential oils remained acceptable even at the end of the storage period, and it was found that cumin essential oil addition to UF-soft cheese gained the highest scores for the sensory attributes^63^. Marjoram and Sage extracts as additives in Kariesh cheese is highly recommended due to their health effect but with a concentration lower than 2%^64^. Sensory characterization of fresh soft cheese fortified with ginger, clove, and thyme oils displayed overall higher acceptability scores than control samples (*p* < 0.001)^26^. Thyme, Moringa, and Cardamom oils improved the sensory parameters and total score points of enriched UF-white soft cheese compared with the control samples and remained acceptable during the storage period^65^.

## Conclusions

The present study established the antibacterial and antibiofilm efficacy of four ethanolic extracts derived from medicinal plants, namely Cinnamon bark, Chamomile flowers, Marigold flowers, and Sage leaves, against both Gram-positive and Gram-negative foodborne pathogenic bacteria. Combining these extracts with low concentrations was more effective for biofilm inhibition than using each extract separately. In order to validate the safety of pasteurized milk and white soft cheese on a small scale (*in* vitro), the tested mixture was used to control *L. monocytogenes* ATCC7646 biofilm on stainless steel surfaces, and it showed great improvement results in produced cheese safety and quality parameters with a significant decrease in *L. monocytogenes* count even during cold storage with more favoured organoleptic parameter.

Consequently, the study's findings suggest that the extracts of Chamomile, Sage, and Cinnamon exhibit antimicrobial and antibiofilm properties without toxicity. Therefore, these extracts could be considered as an alternative or complementary approach to mitigate infections caused by *L. monocytogenes*. Future research on this combination should examine the molecular mechanism as well as the virulence factors of Listeria on a large scale (*In* vivo).

### Supplementary Information

Supplementary Figures.

## Data Availability

The authors declare that the article and its supplementary information contain all the data established and analyzed during this investigation.
