# Supplementary material for: Elimination of detached Listeria monocytogenes from the biofilm on stainless steel surfaces during milk and cheese processing using natural plant extracts
Source: Sci Rep. 2024 Jan 27;14:2288. doi: 10.1038/s41598-024-52394-9 (PMC10821901; doi:10.1038/s41598-024-52394-9)
Supplement: Supplementary file 1 — Supplementary Figures. [file 41598_2024_52394_MOESM1_ESM.docx]

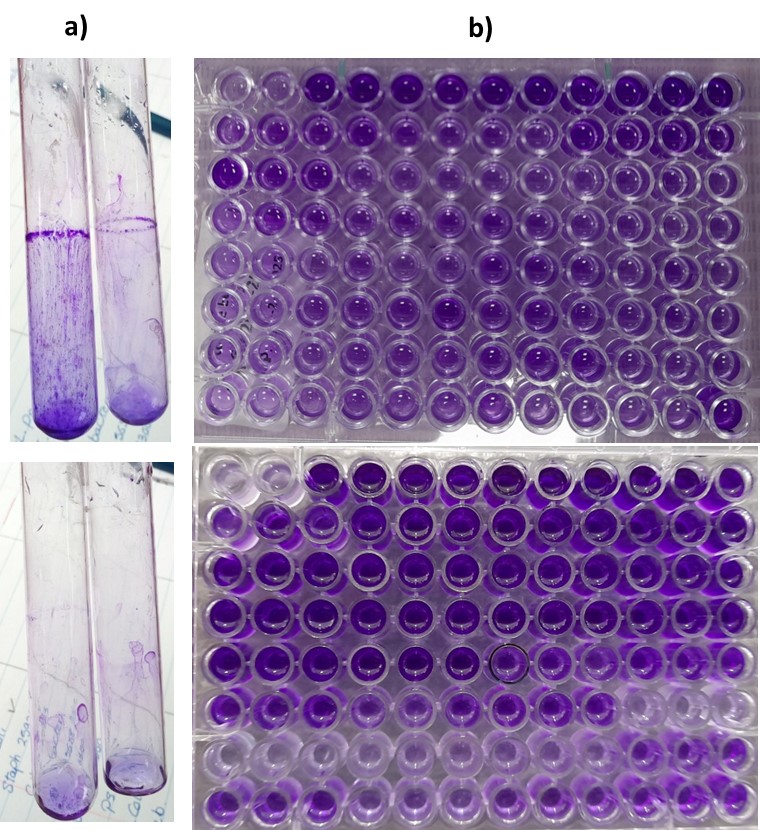


**Fig. S1. Antibiofilm of plant extracts against planktonic cells of pathogenic bacteria.**

**a) Test tube**

**b) Microtiter plate.**

**Fig. S2. The model graph for the biofilm inhibition percentage of plant extracts against planktonic cells of *L. monocytogenes* at a one-component level using L-optimal mixture design.**

A: Chamomile, B: Sage, C: Cinnamon and D: Antibiotic.

**Fig. S3. The model graph for the biofilm inhibition percentage of plant extracts against planktonic cells of *L. monocytogenes* at the interaction of the two-component level using L-optimal mixture design.**

A: Chamomile, B: Sage, C: Cinnamon and D: Antibiotic.
